# Practical media formulations for rapid growth of *Lactobacillus iners* and other vaginal bacteria

**DOI:** 10.1128/aem.00183-25

**Published:** 2025-07-08

**Authors:** Daniella Serrador, Jhenielle R. Campbell, Landon J. Getz, Dorothy Cheung, Gelila Shefraw, Rupert Kaul, William Wiley Navarre

**Affiliations:** 1 Department of Molecular Genetics, University of Toronto 7938, Toronto, Ontario, Canada; 2 Department of Biochemistry, University of Toronto 7938, Toronto, Ontario, Canada; 3 Department of Medicine, University of Toronto 7938, Toronto, Ontario, Canada; 4 Department of Immunology, University of Toronto 7938, Toronto, Ontario, Canada; 5 Toronto General Hospital Research Institute, University Health Network 7989, Toronto, Ontario, Canada; University of Illinois Urbana-Champaign, Urbana, Illinois, USA

**Keywords:** culture conditions, culture medium, vaginal microbiota, *Lactobacillus iners*, liquid culture media, *Gardnerella vaginalis*

## Abstract

The composition of the vaginal microbiome is closely tied to host health. Bacterial vaginosis (BV), caused by the overgrowth of specific anaerobes (e.g., *Gardnerella vaginalis*), is associated with negative health outcomes. A vaginal microbiome dominated by *Lactobacillus* species is thought to protect against BV. However, the role of *Lactobacillus iners* is controversial, with evidence suggesting that some strains may not protect against BV while others do. To better characterize *L. iners* strains, their interactions with vaginal bacteria and human cells need to be investigated *in vitro*, but this has been impeded by the lack of liquid media that supports rapid *L. iners* growth. We have developed three liquid media formulations for *L. iners* growth: Serrador’s *Lactobacillus*-adapted Iscove’s medium (SLIM), which supports robust *L. iners* growth; a vaginally adapted version of SLIM (SLIM-V); and a chemically defined version (SLIM-CD). SLIM and SLIM-V improve *L. iners* growth compared to previously published formulations and also support the growth of other vaginal bacteria, including *Lactobacillus crispatus*, *Lactobacillus jensenii*, *Lactobacillus gasseri*, and *Gardnerella vaginalis*. SLIM-CD leads to slower growth but may be useful for characterizing *L. iners* nutrient requirements or metabolite production. Importantly, SLIM and SLIM-V also support the growth of human vaginal epithelial cells, providing a foundation for future co-culture studies. Here, we present the formulations of SLIM, SLIM-V, and SLIM-CD and compare the growth of bacterial strains and human cells in these media.

**IMPORTANCE:**

*Lactobacillus iners* is one of the most prevalent members of the vaginal microbiome, but whether it promotes health or leads to bacterial vaginosis is not well understood. We have developed media formulations that lead to improved *L. iners* growth and support growth of other vaginal bacteria and human vaginal cells. This will allow for investigation of how *L. iners* interacts with vaginal bacteria and the host, improving our understanding of its role in the vaginal microbiome.

## INTRODUCTION

The composition of the vaginal microbiome greatly impacts host health. Colonization by *Lactobacillus* species, such as *Lactobacillus crispatus*, is considered optimal, while colonization by other anaerobes, like *Gardnerella* and *Prevotella* species, can lead to bacterial overgrowth, resulting in bacterial vaginosis (BV) (1). BV is the most common cause of vaginal complaints among reproductive-age women, affecting an estimated 23%–29% globally (2). In addition to unpleasant vaginal symptoms, BV is associated with increased risk of preterm delivery, low birth weight, cervical cancer, and acquisition of several sexually transmitted infections, including HIV and herpes (3–7). After standard antibiotic treatment, BV will recur in over 50% of patients (8). A better understanding of the dynamics of the vaginal microbiome is needed to improve treatment and prevention efforts.

*Lactobacillus* species are thought to protect against BV by directly or competitively inhibiting growth of BV-associated bacteria (BVAB) (9). A significant proportion of vaginal microbiomes are dominated by one of four *Lactobacillus* species: *Lactobacillus crispatus*, *Lactobacillus iners*, *Lactobacillus gasseri*, or *Lactobacillus jensenii*, with the former two being more prevalent (10–13). While *L. iners* is estimated to be the most common vaginal bacteria globally, its role in vaginal health is controversial, with some labeling *L. iners*-dominated microbiomes as a “transitional” state, between the other “healthy” *Lactobacillus* and BV (14–16). Unlike other vaginal *Lactobacillus*, *L. iners* is frequently present in the vagina along with BVAB during BV (11). *L. iners*-dominated microbiomes transition to a BV-like microbiome, termed molecular BV, more frequently on average than those dominated by the other *Lactobacillus* sp., though some *L. iners*-dominated women are stably colonized by *L. iners* while others have frequent transitions to molecular BV (17, 18). Additionally, *L. iners* produces a pore-forming toxin, inerolysin, capable of lysing human epithelial cells, which has elevated expression in patients with BV (19, 20).

Several groups have proposed that different strains of *L. iners* may differ in their ability to protect against BV, perhaps through differences in their ability to inhibit BVAB (14–16). For example, some *L. iners* strains encode a lanthipeptide (inecin L), capable of inhibiting the growth of *Gardnerella vaginalis* when expressed in *Escherichia coli*. (21) While the interactions of other vaginal *Lactobacillus* species with BVAB have been explored, *L. iners* is frequently omitted (22, 23). One study examining interactions of *Lactobacillus* and BVAB in co-culture included only a single *L. iners* strain, and another study comparing how supernatant from strains of *Lactobacillus* impacted *G. vaginalis* and *Prevotella bivia* growth excluded *L. iners* completely (22, 23). The paucity of experimental data on *L. iners* is largely due to the lack of liquid media formulations that reliably allow robust *L. iners* growth.

*L. iners* does not grow in de Man-Rogosa-Sharpe (MRS) broth, the standard cultivation media for *Lactobacillus* species (24, 25). Recently, Bloom et al. (26) published a medium for *L. iners* growth that consisted of MRS with additional cysteine and glutamine (MRS-CQ). In MRS-CQ, they found *L. iners* growth to maximum density required 36 hours of incubation (26). MRS is composed of beef extract, yeast extract, and peptone, a tryptic meat digest, components that vary by batch or manufacturer (25). Indeed, the ability of *L. iners* to grow in MRS-CQ was found to differ depending on the manufacturer of the base MRS media (26).

To facilitate further *in vitro* characterization of *L. iners*, we have developed three inexpensive and easy-to-make liquid media formulations for improved *L. iners* growth. Serrador’s *Lactobacillus*-adapted Iscove’s medium (SLIM) leads to rapid and robust growth of several *L. iners* strains, as well as vaginal *Lactobacillus* and *Gardnerella* strains. *L. iners* growth is slightly improved by a vaginally adapted medium (SLIM-V). SLIM and SLIM-V can be used for co-culture of bacteria with vaginal epithelial cells. Finally, a fully chemically defined medium (SLIM-CD) grows several *L. iners* strains and will prove useful for metabolite characterization or analyzing the impact of specific nutrients on *L. iners* growth.

## MATERIALS AND METHODS

### Media preparation

To create MRS-CQ, MRS broth (BD Difco, Franklin Lakes, NJ, 288130) was prepared according to manufacturer’s instructions. L-cysteine and L-glutamine (see Table 1) were added at concentrations used by Bloom et al*.* (26) (4 mM and 1.1 mM, respectively). Media was passed through a 0.2 µm filter (Nalgene, Thermo Fisher, Waltham, MO, 595-5420) and stored at 4°C until use. We found that the addition of cysteine and glutamine to MRS from suppliers other than BD-Difco did not reliably support the growth of *L. iners* (Fig. S1).

**TABLE 1:** Formulations for SLIM, SLIM-V, and SLIM-CD^*a*^

Component	SLIM	SLIM-V	SLIM-CD	Concentration (mM)	Supplier	Catalog no.
Liquid base						
IMDM, no phenol red	989 mL	989 mL	**889** mL	–	Gibco	21056-023
Trace mineral supplement	10 mL	10 mL	10 mL	–	ATCC	MD-TMS
TWEEN 80	1 mL	1 mL	1 mL	–	BioShop	TWN507.100
10× nucleotide stock	–	–	100 mL	–	See Table 2	See Table 2
Amino acid source						
Meat peptone	10 g	10 g	–	–	Criterion	C7481
L-Cysteine-HCl	0.63 g	0.63 g	0.63 g	4.0	Sigma	30120-50G
L-Glutamine	0.16 g	0.16 g	0.16 g	1.1	BioShop	GLU102.25
L-Glutamic acid	–	–	2.02 g	13.7	BioShop	GLU202.100
L-Proline	–	–	1.02 g	8.9	BioShop	PRO222.25
L-Leucine	–	–	0.83 g	6.3	Sigma	L8912-25G
L-Lysine-HCl	–	–	0.74 g	4.1	Bio Basic	LB0171
L-Valine	–	–	0.65 g	5.5	BioShop	VAL201.25
L-Aspartic acid	–	–	0.64 g	4.8	BioShop	ASP003.25
L-Serine	–	–	0.57 g	5.4	Aldrich	S2600-10G
L-Tyrosine	–	–	0.57 g	3.1	BioShop	TYR333.50
L-Isoleucine	–	–	0.55 g	4.2	BioShop	ISO910.10
L-Phenylalanine	–	–	0.45 g	2.7	Bio Basic	PB0422
L-Threonine	–	–	0.44 g	3.7	BioShop	THR002.25
L-Arginine-HCl	–	–	0.37 g	1.8	BioShop	ARG006.100
L-Histidine-HCl	–	–	0.28 g	1.5	Bio Basic	HB0330
L-Alanine	–	–	0.27 g	3.0	BioShop	ALA001.25
L-Methionine	–	–	0.25 g	1.7	Sigma	M9625-100G
Glycine	–	–	0.24 g	3.2	Sigma	G8898-1KG
L-Tryptophan	–	–	0.11 g	0.5	BioShop	TRP100.25
Vaginal adaptation						
Urea	–	0.4 g	0.4 g	6.7	BioShop	URE001.500
Acetic acid	–	960 µL	960 µL	–	Caledon	1000-1-29
Glycerol	–	128 µL	128 µL	–	BioShop	GLY002.4

^*a*^ Recipes to create 1 L of media. – indicates component not added or concentration (mM) not applicable. When a component’s amount differs between formulations, values are bolded and underlined.

SLIM is created by adding components to Iscove’s modified Dulbecco’s medium (IMDM), a chemically defined, commercially available cell culture medium (27). To create SLIM, IMDM is supplemented with 10 g/L meat peptone, 10 mL/L ATCC trace mineral supplement, 4 mM L-cysteine, and 1.1 mM L-glutamine (see Table 1). SLIM also contains 0.1% TWEEN 80 unless being used for co-culture experiments with mammalian cells (Table 1). SLIM-V was developed by adding urea, glycerol, and acetic acid at concentrations used in a simulated vaginal fluid developed by Owen and Katz (28) (Table 1; Table S1). SLIM-CD was developed by replacing the peptone in SLIM-V with a nucleotide supplement (Table 2) and additional amino acids (Table 1). After preparation, media was passed through a 0.2 µm filter (Nalgene, Thermo Fisher, Waltham, MO, 595-5420) and stored at 4°C until use.

**TABLE 2:** Composition of nucleotide stock^*a*^

Compound	Amount	Concentration (mM)	Supplier	Catalog no.
ddH_2_O	498.75 mL	–	–	–
Potassium hydroxide	0.421 g	7.5	BioShop	PHY202.500
Guanine	0.151 g	1.0	BioShop	GUA904.5
Adenine HCl	0.135 g	0.8	Sigma	A8751-5G
Uracil	0.112 g	1.0	Sigma	U-0750
Cytosine	0.111 g	1.0	BioShop	CYT301.5

^*a*^ Recipe for 500 mL of 10× stock. – indicates not applicable. Concentrations based on 10× ACGU solution (TekNova, Hollister, CA).

The 10× nucleotide stock was prepared by dissolving components in ultra-pure water and then passing the solution through a 0.2 µm filter (Nalgene, Thermo Fisher, Waltham, MO, 595-5420). The stock was stored at −20°C until use. Concentrations were based on 10X ACGU solution (TekNova, Hollister, CA) (Table 2).

### Bacterial cultivation

All bacterial cultivation and growth assays were performed in an anaerobic chamber (AS-580, Anaerobe Systems, Morgan Hill, CA) using a gas mixture of 10% CO_2_, 10% H_2_, and balance N_2_ (Linde, Mississauga, ON). Liquid media was allowed to deoxygenate in the chamber for at least 24 hours prior to use, and agar plates were deoxygenated for at least 7 hours prior to use. *L. iners* and *Gardnerella* species were grown on New York City III (NYC III, see Table S2) agar plates (1.5% wt/vol agar, BioShop, Burlington, ON, AGR001.500) (29). All other lactobacilli, including vaginal *Lactobacillus*, were grown on MRS (NutriSelect Basic, Sigma-Aldrich, St. Louis, MO, 69966-500G) agar plates (1.5% wt/vol agar, BioShop, Burlington, ON, AGR001.500). Bacterial stocks were maintained at −80°C in brain-heart infusion broth (Millipore, Darmstadt, Germany, 75917-500G) with 20% glycerol (BioShop, Burlington, ON, GLY002.4). After streaking frozen stocks onto plates, plates were incubated at 37°C for 48–66 hours.

### Bacterial strains

Experiments included previously published strains of *L. iners*, *L. crispatus*, *L. gasseri*, *L. jensenii*, and *G. vaginalis*, as well as six *L*. *iners* isolates, one *L*. *crispatus* isolate, one *L*. *gasseri* isolate, and one *Gardnerella* isolate cultivated from cervicovaginal secretions (Table 3). Cervicovaginal secretion samples were provided by the lab of Dr. Rupert Kaul (Immunology, University of Toronto). In brief, female sex workers in Nairobi, Kenya, were recruited through the Sex Worker’s Outreach Program clinics, and they were provided informed, written consent for immune and microbial studies (REB approval in protocol #37046). Consenting participants provided cervicovaginal secretions collected by SoftCup (Evofem, San Diego, CA). Secretions were diluted 10-fold in sterile phosphate buffered saline (PBS), then centrifuged at 1730 × *g* for 10 minutes, after which supernatant was extracted and pellets were resuspended in 500 µL PBS. Both were frozen at −80°C and transported to the Kaul lab at the University of Toronto for analysis.

**TABLE 3:** Vaginal bacterial strains used in this study

Species	Strain	Source	Original study
*L. iners*	AB107 (ATCC 55195)	Human vagina	Allen, 1992 (30)
*L. iners^a^*	SPIN 2503V10-D (BEI HM-704)	Patient with BV	BEI/Human Microbiome Project
*L. iners*	NVM025S01	Cervicovaginal secretions	This study
*L. iners*	NVM020S14	Cervicovaginal secretions	This study
*L. iners*	NVM210S01	Cervicovaginal secretions	This study
*L. iners*	NVM196S05	Cervicovaginal secretions	This study
*L. iners*	NVM041S27	Cervicovaginal secretions	This study
*L. iners*	NVM076S08	Cervicovaginal secretions	This study
*L. gasseri^a^*	EX336960VC01 (BEI HM-398)	Female urogenital tract	BEI/Human Microbiome Project
*L. gasseri*	NVM088S01	Cervicovaginal secretions	This study
*L. crispatus^a^*	PSS7772C (BEI HM-1277)	Urine of a pregnant woman	Human Microbiome Project
*L. crispatus*	NVM146S01	Cervicovaginal secretions	This study
*L. jensenii^a^*	269-3 (BEI HM-645)	Human vaginal mucosa	BEI/Human Microbiome Project
*L. jensenii^a^*	EX849587VC03 (BEI HM-372)	Human mid-vaginal wall	BEI/Human Microbiome Project
*G. vaginalis*	ATCC 14019	Vaginal secretions	Gardner and Dukes, 1954 (31)
*Gardnerella* sp.	NVM022C02	Cervicovaginal secretions	This study

^*a*^ These isolates were obtained through BEI Resources, NIAID, NIH, as part of the Human Microbiome Project (https://commonfund.nih.gov/human-microbiome-project-hmp).

To isolate bacteria from the cervicovaginal secretions, the pellets were thawed and 10-fold serial dilutions in sterile 1× PBS were created. Dilutions were spread onto agar plates (NYC III for *L. iners* and *Gardnerella*, MRS for *L. crispatus* and *L. gasseri*) and incubated for 48 hours at 37°C. Single colonies were picked and restruck onto agar plates and then incubated for 48 hours at 37°C. This was repeated twice, at which point colony morphology for each isolate was consistent, and stocks were frozen.

The species of each isolate was identified using PCR with species-specific primers (Table S3). For the *L. iners* isolates, isolate gDNA was extracted from 2 mL of culture grown in SLIM for 42 hours using the Wizard Genomic DNA Purification Kit (Promega, Madison, WI, USA, A1120). Genome sequencing was performed using the Oxford Nanopore Flongle platform (Oxford Nanopore, Oxford, UK). In brief, genomic DNA libraries were generated using the Rapid Barcoding Kit 24 V14 (Oxford Nanopore, Oxford, UK). Base calling and genome assembly was performed using Dorado’s super accurate model (Oxford Nanopore, Oxford, UK) and Flye version 2.9.3-b1797, respectively (32). Average nucleotide identity (ANI) between *L. iners* isolates was determined using the EZBioCloud ANI calculator (33). The *L. iners* isolates were determined to be unique strains as ANI was <99.5% between them (34), except between NVM196S05 and NVM210S01. These isolates were identified as different strains, as NVM210S01 encodes a glycerol catabolism operon (*glpKO*) that NVM196S05 lacks.

### Bacterial growth measurements

For *L. iners*, growth in SLIM, SLIM-V, SLIM-CD, and MRS-CQ was compared. In the anaerobic chamber, for each media type, three 13 mL polypropylene tubes (Sarstedt, Nümbrecht, Germany, 62.515.006) per bacterial strain tested were prepared with 4 mL of the deoxygenated media. Tubes were inoculated with two to three bacterial colonies from fresh agar plates and then incubated anaerobically at 37°C. At each time point, cultures were removed from the 37°C incubator but remained in the anaerobic chamber. Cultures were resuspended, as without shaking, cells settle to the bottom of the tubes during incubation, and 200 µL from each culture, as well as media blanks, was aliquoted into a 96-well plate (Sarstedt, Nümbrecht, Germany, 83.3924), after which cultures were returned to the incubator. The 96-well plate was removed from the anaerobic chamber, and optical density at 600 nm (OD_600_) was measured using an Infinite M Nano+ (Tecan, Männedorf, Switzerland).

For growth of other vaginal bacteria, SLIM and SLIM-V were compared to the standard growth media of MRS broth (NutriSelect Basic, Sigma-Aldrich, St. Louis, MO, 69966-500G) for *Lactobacillus* and NYC III broth (Table S2) for *Gardnerella*. Measurements were performed as for the *L. iners* growth curve but with only a single time point after 24 hours of incubation.

### Cell culture and viability assays

The vaginal epithelial cell line VK2/E6E7 (35) was maintained in keratinocyte serum-free media (KSFM) supplemented with 0.05 mg/mL bovine pituitary extract and 0.1 ng/mL human recombinant EGF (Gibco, Waltham, MO, 17005042). The complete keratinocyte serum-free media was additionally supplemented with 44.1 mg/L calcium chloride. Cells were grown at 37°C in 5% CO_2_ with media replacement every 2–3 days and passaged weekly. The viability of the VK2 cells in the developed media was tested using two methods, trypan blue exclusion and a metabolic resazurin assay, where the fluorescence of metabolized resazurin (resorufin) is indicative of the number of viable cells (36). The vaginal cells were harvested using a standard trypsinization protocol and passaged in KSFM into a 96-well plate (200 µL/well) for the resazurin assay or 24-well plate (500 µL/well) for trypan blue exclusion. Seeding densities were 5 × 10^4^ cells/mL and 2 × 10^5^ cells/mL, respectively. Following 2 days of growth to 90%–95% confluency, the media was removed from the wells, and the adherent cells were washed with 1× PBS. Experimental media (SLIM, SLIM-V, and SLIM-CD) and control media (KSFM) were aliquoted into the appropriate wells.

Cell viability for VK2 cells was enumerated using the trypan blue assay following the 3 hour incubation in the tested media formulations. Adherent vaginal cells were washed with PBS and dislodged using 0.25% Trypsin-EDTA (Gibco, Waltham, MO, USA, 17005042). Trypsin was neutralized by addition of Dulbecco's modified Eagle medium (DMEM)/F12 supplemented with 10% fetal calf serum (Gibco, Waltham, MO, USA, 17005042). Live cells that exclude the dye and dead cells that uptake the dye were counted using a hemocytometer after mixing (1:1) the cell suspension with trypan blue. Cell viability is expressed as [number of live cells/total number of live and dead cells] × 100%. In both types of cell viability assays, SLIM media formulations that excluded the addition of TWEEN 80 were also tested on the cells in consideration of the reported cytotoxicity of this detergent on mammalian cells (37).

For the resazurin assay, a set of wells without cells were reserved as media-only controls to measure background fluorescence. After a 3 hour incubation at 37°C in 5% CO_2_, the cells were imaged using a light microscope (Evos FLoid, Thermo Fisher, Waltham, MO, USA, 4471136) before 40 µL of a 0.15 mg/mL resazurin solution was added to each well and incubated for 3 hours. Cytation 5 Microplate Reader (Agilent BioTek, Santa Clara, CA, USA, CYT5MFA) was used to determine metabolic cell viability by measuring fluorescence at 560 nm excitation and 590 nm emission. Parallel resazurin assays were also conducted on the human endometrial carcinoma cell line (HEC-1-B). HEC-1-B cells were maintained in DMEM (Wisent, Saint-Jean-Baptiste, QC, Canada, 319-005-CL) supplemented with 10% fetal bovine serum (Gibco, Waltham, MO, USA, 12483-020) and 1% penicillin-streptomycin (Gibco, Waltham, MO, USA, 15140-122).

## RESULTS

### *L. iners* growth in SLIM formulations

**Fig 1:**
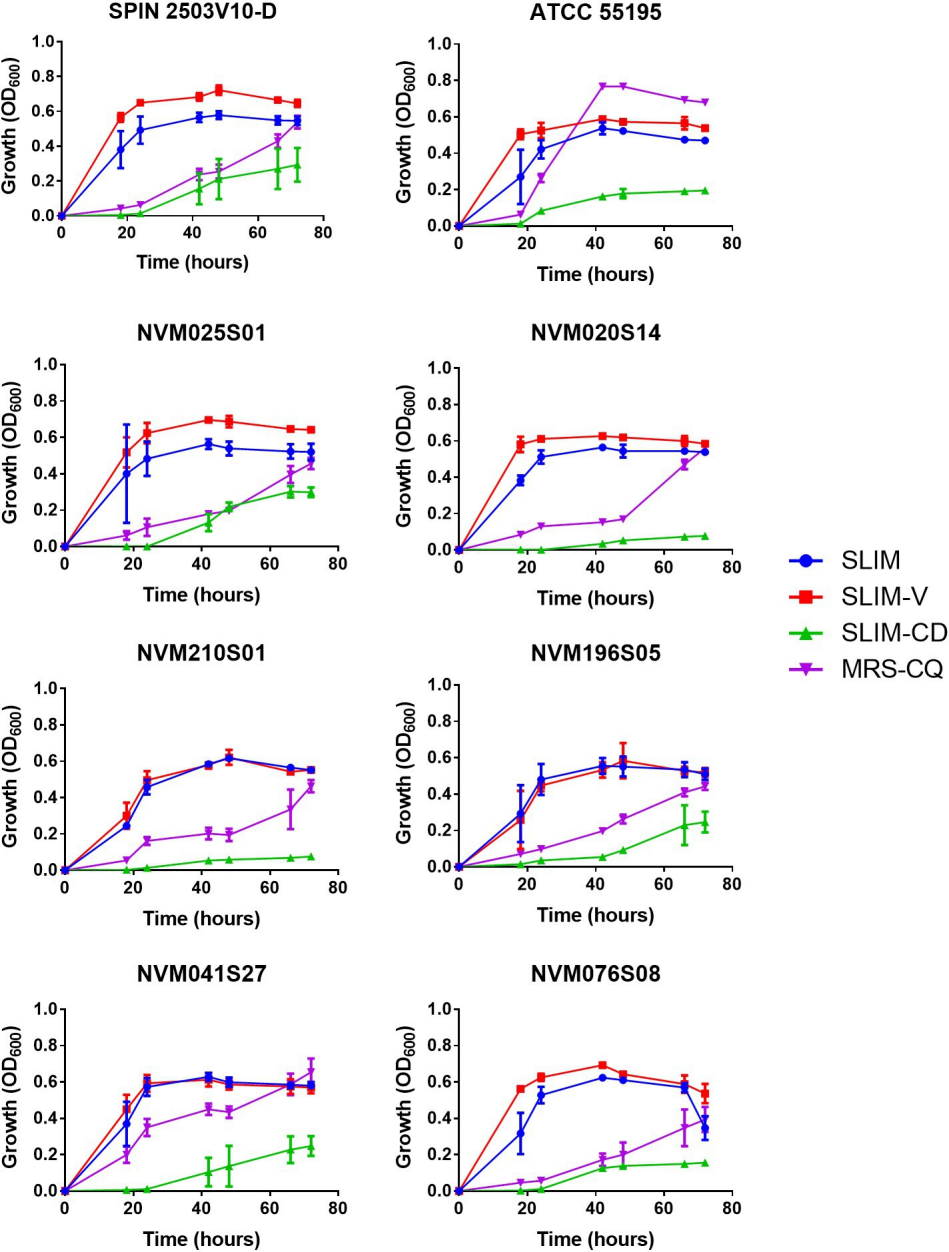
Growth of *L. iners* strains in SLIM, SLIM-V, SLIM-CD, and MRS-CQ. Colonies of *L. iners* were inoculated into 4 mL of the indicated liquid growth media. OD_600_ measured at the indicated time points using an Infinite M Nano+ (Tecan, Männedorf, Switzerland), with average value of 3 media blanks subtracted. *n* = 3, mean ± SD. Representative results from 1 of ≥2 independent experiments.

We tested whether modifications of IMDM, a standard tissue culture media, could support the growth of vaginal lactobacilli including *L. iners*. This line of inquiry was based on our observation that several fastidious lactobacilli grew well when stationary phase cultures in MRS were diluted (subcultured) into fresh IMDM (Fig. S2A). Growth was not observed if bacterial colonies were inoculated directly into IMDM but was observed when colonies were inoculated into IMDM with 1% MRS (Fig. S2A), suggesting that some component present in MRS, when added to IMDM, was supporting bacterial growth. Addition of a trace mineral supplement to IMDM resulted in growth of *Lactiplantibacillus plantarum*, though not other lactobacilli (Fig. S2B). Addition of TWEEN 80, a component of MRS, led to more rapid *L. plantarum* growth (Fig. S2C). We then added additional MRS components to IMDM with the mineral supplement, testing yeast extract, meat extract, and peptone, and found that IMDM with peptone and the trace mineral supplement resulted in superior growth of *L. plantarum* and *Lacticaseibacillus rhamnosus* (Fig. S2D). Combining IMDM with peptone, TWEEN 80, and the mineral supplement supported growth of a variety of lactobacilli, including several vaginal strains (Fig. S2E). Subsequent addition of cysteine and glutamine supported growth of several isolates of *L. iners* (Fig. 1). This modified IMDM was designated SLIM. We aimed to support growth of a variety of *L. iners* strains, and thus, SLIM is not an optimized minimal media. SLIM-V is a SLIM media further modified with urea, acetate, and glycerol: compounds found in vaginal fluid. We observed that *L. iners* grew in SLIM-V when peptone was replaced by a nucleotide supplement (Table 2) and casamino acids (Bio Basic, Markham, ON, Canada, CB3060), an acid-hydrolysis of the milk protein casein (Fig. S3). To create a chemically defined medium (SLIM-CD), we replaced casamino acids with an amino acid mixture matching the composition of casein supplements as reported by Rasmussen et al. (38) (Table 1).

We tested the ability of SLIM and SLIM-V to support the growth of a panel of eight unique *L. iners* isolates (Fig. 1). For all *L. iners* strains tested, growth was significantly greater in SLIM and SLIM-V than in MRS-CQ at 18 and 24 hours. After 42 hours, *L. iners* ATCC 55195 had increased growth in MRS-CQ compared to SLIM and SLIM-V, while the seven other strains had stronger growth in SLIM formulations until growth in MRS-CQ caught up at 66 or 72 hours (Fig. 1). 5/8 strains had slightly improved growth in SLIM-V compared to in SLIM, while the remaining 3/8 strains had no difference between growth in SLIM and SLIM-V (Fig. 1). SLIM-CD led to light growth of six strains, some requiring 48 hours of incubation while others required 66 hours (Fig. 1). Two strains that grew reasonably well in SLIM (NVM020S14 and NVM210S01) did not reach our light growth threshold of OD_600_ values > 0.1 in SLIM-CD (Fig. 1). As relative growth in SLIM vs SLIM-V and SLIM vs MRS-CQ varied by strain and several strains did not grow in SLIM-CD, different *L. iners* strains have different nutrient preferences.

### Growth of other bacteria in SLIM

**Fig 2:**
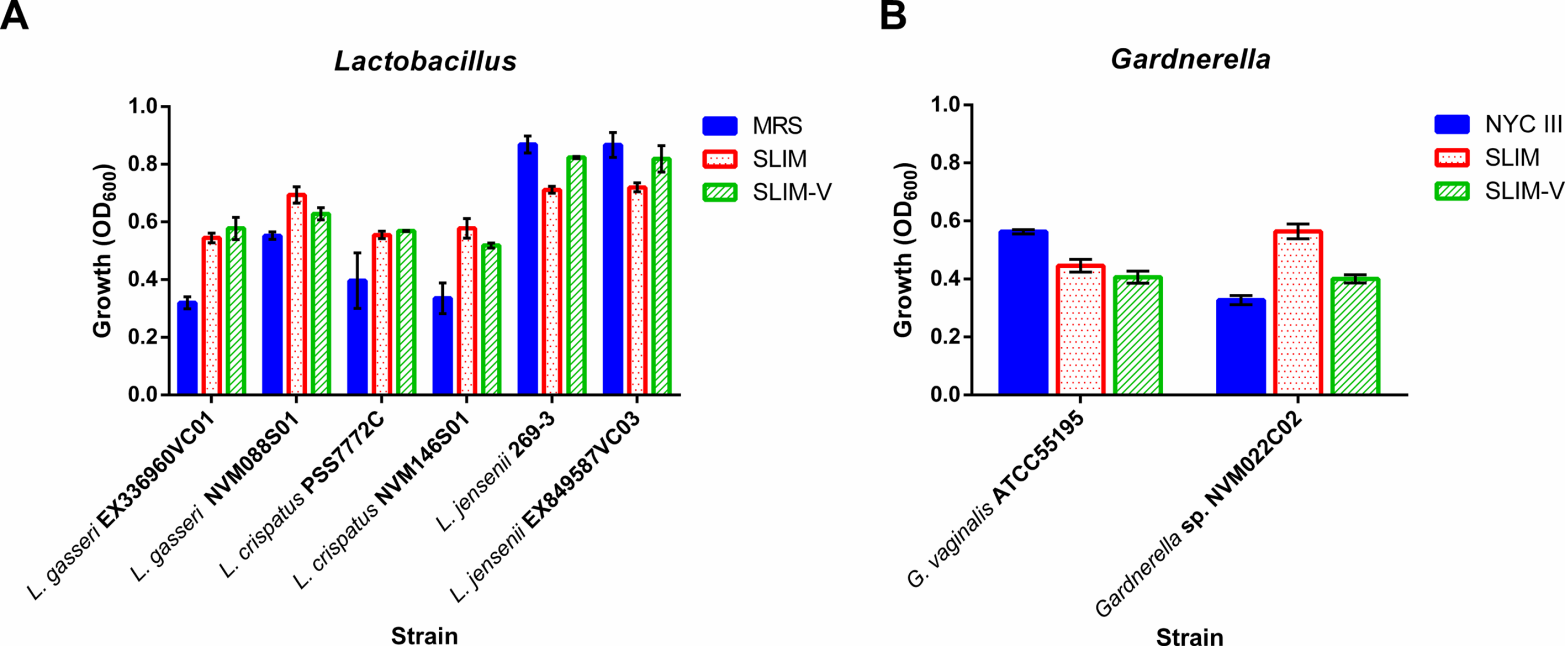
Growth of vaginal *Lactobacillus* (**A**) and *Gardnerella* (**B**) strains in SLIM and SLIM-V. Growth of various vaginal lactobacilli and *Gardnerella* sp. measured after 24 hours of incubation at 37°C. OD_600_ measured using an Infinite M Nano+ (Tecan, Männedorf, Switzerland), with average value of 3 media blanks subtracted. *n* = 3, mean ± SD. Representative results from 1 of ≥2 independent experiments.

We tested SLIM and SLIM-V for their ability to support growth of other vaginal bacteria including two strains of *L. gasseri*, *L. crispatus*, and *L. jensenii*, as well as one strain of *G. vaginalis* and one isolate of an unclassified *Gardnerella* species (Fig. 2). For both *L. gasseri* and *L. crispatus* strains tested, as well as *Gardnerella* sp. NVM022C02, growth in SLIM and SLIM-V was improved compared to their standard growth media of MRS or NYC III, respectively (Fig. 2). Some strains grew better in SLIM-V than SLIM (both *L. jensenii* strains), while *Gardnerella* sp. NVM022C02 preferred SLIM to SLIM-V (Fig. 2). The remaining strains had similar growth in SLIM and SLIM-V (Fig. 2).

We also find that SLIM supports growth of several non-vaginal lactobacilli, including *L. plantarum*, *L. rhamnosus*, *Limosilactobacillus reuteri*, *Ligilalactobacillus murinus*, *Ligilalactobacillus ruminis*, *Logiolactobacillus coryniformis*, *Companilactobacillus farciminis*, *Lactobacillus intestinalis*, *Lactobacillus psittaci*, and *Lactobacillus johnsonii* (Table S4; Fig. S4).

### Viability of human epithelial cells in SLIM

**Fig 3:**
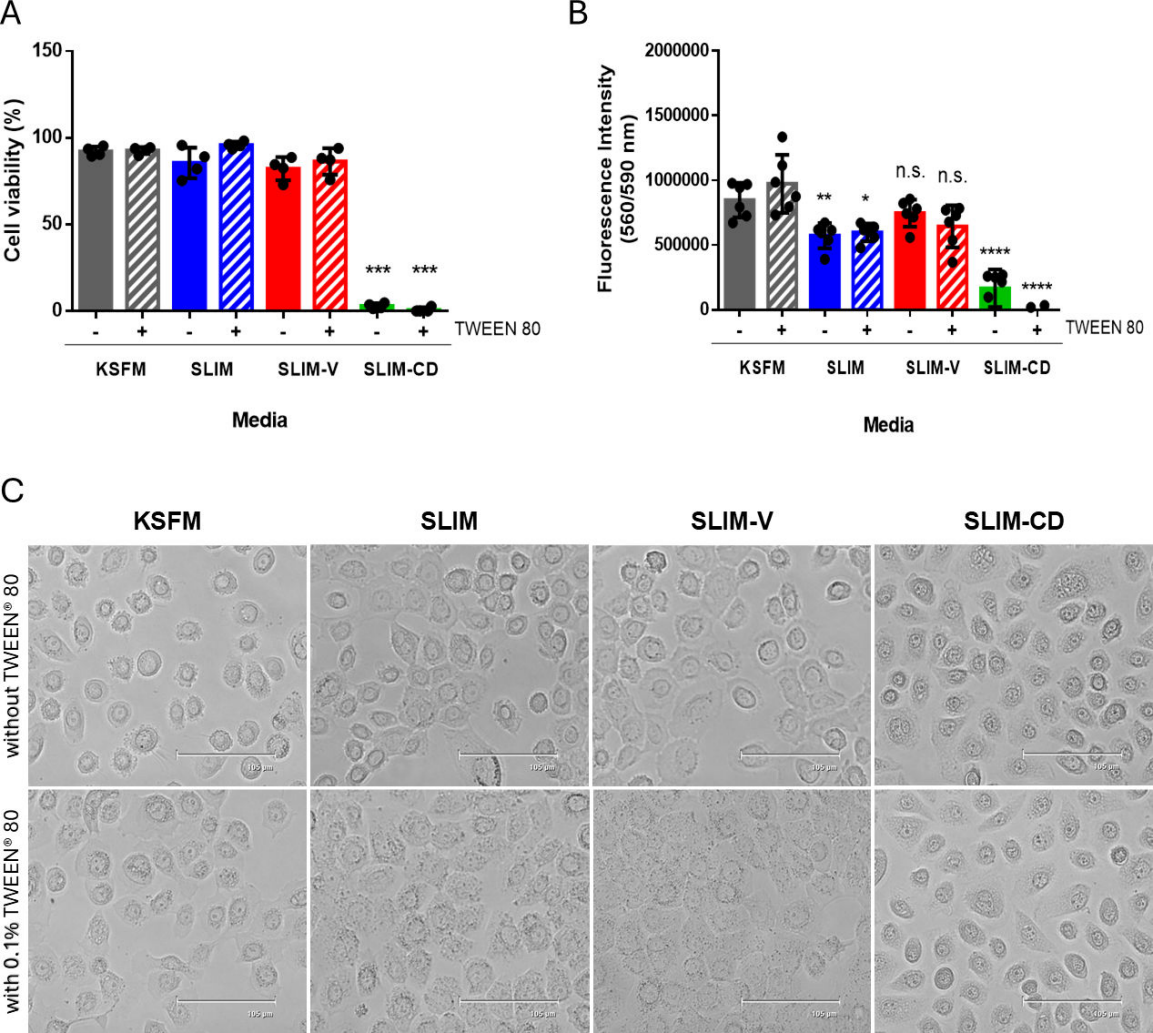
Viability of VK2 vaginal epithelial cells in SLIM formulations for 3 hours. (**A**) Trypan blue exclusion assay enumerating cell viability. Cell viability calculated as [number of live cells/total number of live and dead cells] × 100%. Mean ± SD; *n* = 4 with the inclusion of two biological replicates from independent experiments. Statistical significance in comparison to KSFM without TWEEN 80 was calculated using one-way analysis of variance, ***P < 0.001. (**B**) Resazurin metabolic measure of cell viability. The fluorescence intensity of resorufin produced by live cells metabolizing resazurin is proportional to cell viability. Background fluorescence of the media-only controls is subtracted from the fluorescence intensity of the appropriate sample wells. Mean ± SD; *n* = 6 with the inclusion of two biological replicates from independent experiments. Statistical significance in comparison to KSFM without TWEEN 80 was calculated using one-way analysis of variance, *P > 0.05, **P < 0.01, and ****P < 0.0001. (**C**) Representative light microscopy images of VK2 cells in varying media. Representative results from one of two independent experiments. Images taken at 20× objective (set to 95% zoom). Scale bar represents 105 µm.

Given that SLIM is based on a tissue culture media, the ability of mammalian cells to remain viable in SLIM formulations for 3 hours was assessed through trypan blue exclusion and the metabolic conversion of resazurin into resorufin. VK2 epithelial cells maintained viability in both SLIM and SLIM-V with or without TWEEN 80 (Fig. 3A). However, resazurin turnover by VK2 cells was reduced in SLIM compared to SLIM-V, indicative of reduced metabolic activity (Fig. 3B). Of the media formulations tested, the metabolic activity of the vaginal cells in SLIM-V with and without TWEEN 80 closely mimicked its metabolism in standard cell culture media (Fig. 3B). VK2 viability and metabolism were significantly lower in SLIM-CD (Fig. 3A and B). Although SLIM and SLIM-V did not affect the viability of VK2 cells, differences in morphology were observed. In KSFM, VK2 cells have a mixed morphology of round and polygonal cells, whereas the cells are mostly polygonal in SLIM and SLIM-V (Fig. 3C). Additionally, in formulations of SLIM and SLIM-V that included TWEEN 80, more vacuoles (small dark dots in the cytoplasm) are observed in the VK2 cells, suggesting that the cells are stressed (Fig. 3C) (39). Like vaginal epithelial cells, uterine epithelial cells were also metabolically viable in SLIM-V, but they maintained metabolic viability in SLIM in contrast to the vaginal epithelial cells (Fig. S5A). As observed in vaginal epithelial cells, the HEC-1-B cells also exhibited reduced resazurin turnover in SLIM-CD (Fig. S5A). In HEC-1-B cells, the inclusion of TWEEN 80 in SLIM and SLIM-V also increased the presence of vacuoles in the cytoplasm (Fig. S5B). Together, these results show that the viability of epithelial cells from the female genital tract in SLIM-V without TWEEN 80 is most comparable to standard growth media.

## DISCUSSION

The role of *L. iners* in the vaginal microbiome is currently poorly understood, in large part due to difficulties in cultivation. Here, we present three media formulations, two leading to robust growth of several *L. iners* strains and other vaginal bacteria and one that is chemically defined for nutrient and metabolite analysis. These are not minimal media containing only the nutrients necessary for growth, as we aimed to grow a range of *L. iners* strains as well as other vaginal bacteria and human cells. The trace mineral supplement could likely be replaced with fewer components, and it is probable that not all amino acids added to SLIM-CD are necessary, especially as IMDM already contains a complete set of amino acids. However, initial tests indicate that *L. iners* strains require multiple additional amino acids for optimal growth in SLIM-CD. When SLIM-CD was prepared with subsets of amino acids (four different groups of amino acids, grouped based on concentration in SLIM-CD), media with these subsets led to reduced growth compared to the complete set of amino acids (Fig. S6). This suggests that at minimum, two amino acids from two of these groups are required for optimal growth in SLIM-CD. As we aim to support growth of diverse *L. iners* strains, we chose not to further simplify the media, but future work focusing on specific *L. iners* strains could likely optimize the formulation.

It is somewhat surprising that *L. iners* growth is improved in SLIM compared to MRS-CQ (Fig. 1), which is a nutritionally richer medium. There are two potential explanations: that a component of MRS is inhibitory to *L. iners* growth and absent from SLIM or that components of SLIM that are absent from MRS lead to improved growth. *L. iners* ATCC 55195 and SPIN 2503V10-D growth in a 1:1 mixture of MRS-CQ and SLIM was similar to growth in SLIM (Fig. S7), suggesting the latter hypothesis is more likely. One possibility is the presence of free amino acids in IMDM, as opposed to the peptides that are abundant in MRS. Bloom et al. (26) suggested *L. iners* has a limited capacity to use L-cysteine from complex sources, which was why additional free L-cysteine needed to be added to MRS to grow *L. iners* in MRS-CQ. However, if this is true, the underlying *L. iners* biology is not understood. Other lactic acid bacteria are known to synthesize proteolytic enzymes to obtain free amino acids, but proteolytic activity of *L. iners* has not been characterized (40). Several peptidases and proteases are present and well conserved in *L. iners* genomes, including the strains used in this work. *In vitro* characterization of *L. iners* proteolytic activity is needed. If *L. iners* is indeed weakly proteolytic and prefers free amino acids over peptides, this could explain why IMDM is a more suitable *L. iners* media base than MRS.

*L. iners* growth in MRS-CQ (Fig. 1) was significantly slower in our experiments than that observed by Bloom et al. (26), where growth was seen in 35 hours. This is likely due to differences in cultivation techniques. Bloom et al. (26)state that *Lactobacillus* growth was “adversely affected by either continuous or intermittent agitation,” and so cultures prepared in parallel were used for their growth quantification, whereas here the same cultures are resuspended at each time point. It is possible that SLIM formulations protect better against the adverse effects of agitation than MRS-CQ. However, we still observe improved growth in SLIM and SLIM-V at our first time point, prior to agitation.

Growth was improved for most *L. iners* strains and vaginal epithelial cells in SLIM-V compared to SLIM (Fig. 1), which was designed to be more like the native vaginal environment. Future work will determine the individual impacts of urea, glycerol, and acetic acid. One component absent from SLIM-V that is present in the vaginal tract is glycogen, which has been linked to *Lactobacillus* presence and improves *L. crispatus* growth (22, 41). Future investigations into the impact of glycogen on *L. iners* will assess growth in SLIM-V with additional glycogen or where glycogen is the primary carbon source instead of glucose. While SLIM-CD grew most *L. iners* strains, growth of *L. iners* NVM020S14 and *L. iners* NVM210S01 was minimal (Fig. 1). More work is necessary to determine why these particular isolates failed to grow in our chemically defined media. The differences between strain growth in different media formulations, with some strains having improved growth in SLIM-V while others do not, and SLIM-CD not supporting growth of all strains, indicate that *L. iners* strains have differing nutrient requirements. If vaginal bacteria differ in their requirements for nutrients that vary between vaginal environments, this could explain why individuals are colonized by different bacterial strains and/or species and why probiotics sometimes fail to colonize. Further characterization of both bacterial nutrient requirements and availability of these nutrients in the vaginal environment is needed.

Co-culture experiments with human and bacterial cells are commonly done in a 3 hour window, and it is essential to use a media that supports the viability of both organisms during this time. Of the media formulations tested, SLIM-V without TWEEN 80 is the most comparable to standard growth media in supporting the viability and metabolic activity of both vaginal (VK2) and uterine (HEC-1-B) epithelial cells. While both SLIM and SLIM-V metabolically support uterine epithelial cells, the vaginal adaptations included in the formulation of SLIM-V provide an increased metabolic benefit for vaginal epithelial cells compared to base SLIM (Fig. S5A; Fig. 3B). SLIM-CD conferred worse viability and lower metabolism in both epithelial cell types (Fig. 3A and B; Fig. S5A). Meat peptone may provide growth factors for cell growth, and its exclusion from SLIM-CD is a plausible reason for the observed cell viability issues. TWEEN 80, which is a poly-ethyl-sorbitan-linked derivative of oleic acid, is reported to damage epithelial cells, so disrupted cell morphology seen in media with TWEEN 80 (Fig. 3C; Fig. S5B) is not entirely unexpected (37). Interestingly, inclusion of TWEEN 80 in SLIM and SLIM-V did not alter VK2 viability and metabolism despite differences in morphology (Fig. 3). This was not the case with HEC-1-B cells, where inclusion of TWEEN 80 in most media formulations led to increased metabolic activity (Fig. S5A), indicating differences between these two female genital tract epithelial cells in their response to TWEEN 80. TWEEN 80 is necessary for *L. iners* growth and is sometimes added to mammalian cell culture media to promote growth, but TWEEN 80 concentrations in SLIM could be lowered to reduce epithelial cell stress if used in short-term co-culture assays. SLIM-V provides a base for future co-culture experiments between vaginal bacteria and epithelial cells as it supports the viability of both epithelial cell types in an environment that more closely mimics the vaginal environment compared to standard cell culture media.

## Data Availability

*L. iners* genomes in this study have been deposited in NCBI (BioProject PRJNA1184769).

## SUPPLEMENTAL MATERIAL

Supplemental material aem.00183-25-s0001.docx Figures S1 to S7 and Tables S1 to S4.

ASM does not own the copyrights to Supplemental Material that may be linked to, or accessed through, an article. The authors have granted ASM a non-exclusive, world-wide license to publish the Supplemental Material files. Please contact the corresponding author directly for reuse.
