# Supplementary material for: Practical media formulations for rapid growth of Lactobacillus iners and other vaginal bacteria
Source: Appl Environ Microbiol. 2025 Jul 8;91(8):e00183-25. doi: 10.1128/aem.00183-25 (PMC12366303; doi:10.1128/aem.00183-25)
Supplement: Supplemental material — Figures S1 to S7 and Tables S1 to S4. [file aem.00183-25-s0001.docx]

**
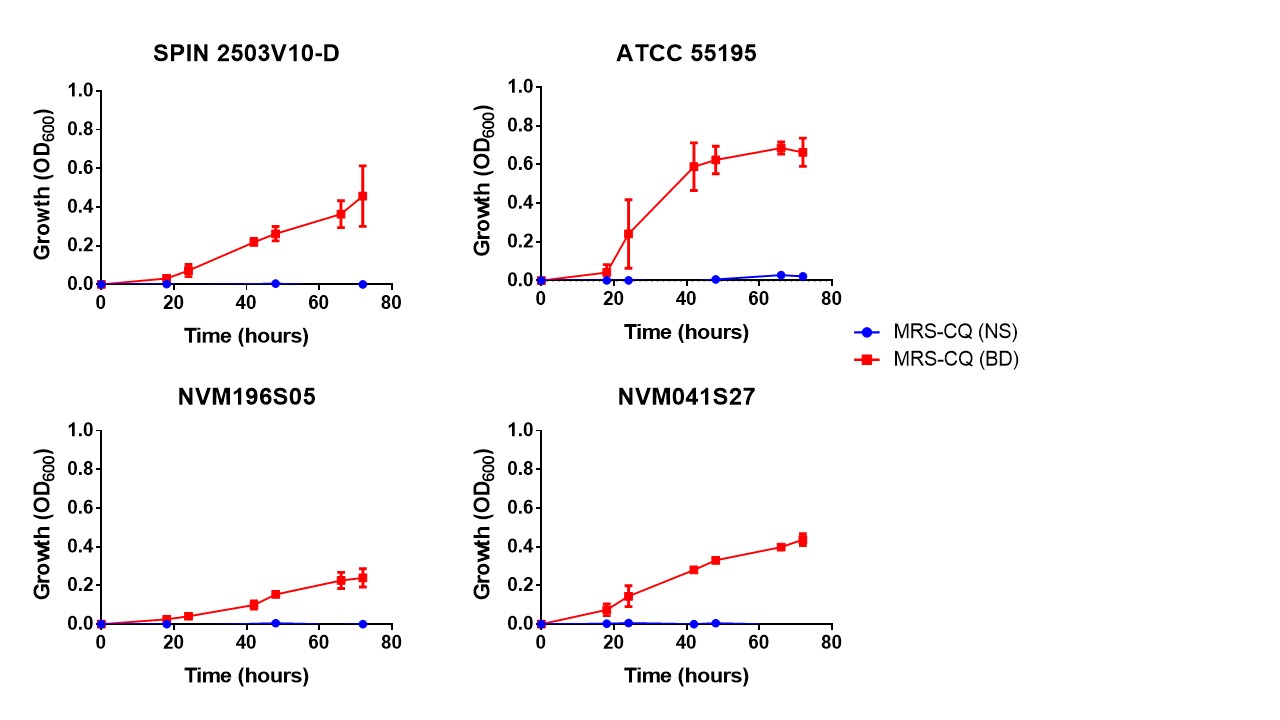
**

**Figure S1: Growth of *L. iners* strains in MRS-CQ with different MRS manufacturers.** MRS-CQ (NS) contains NutriSelect® Basic MRS (Sigma-Aldrich, St. Louis, MO, 69966-500G), while MRS-CQ (BD) contains BD Difco MRS (BD Difco, Franklin Lakes, NJ, 288130). OD_600_ measured using an Infinite® M Nano+ (Tecan, Männedorf, Switzerland), with average value of 3 media blanks subtracted. n = 3, mean ± SD.


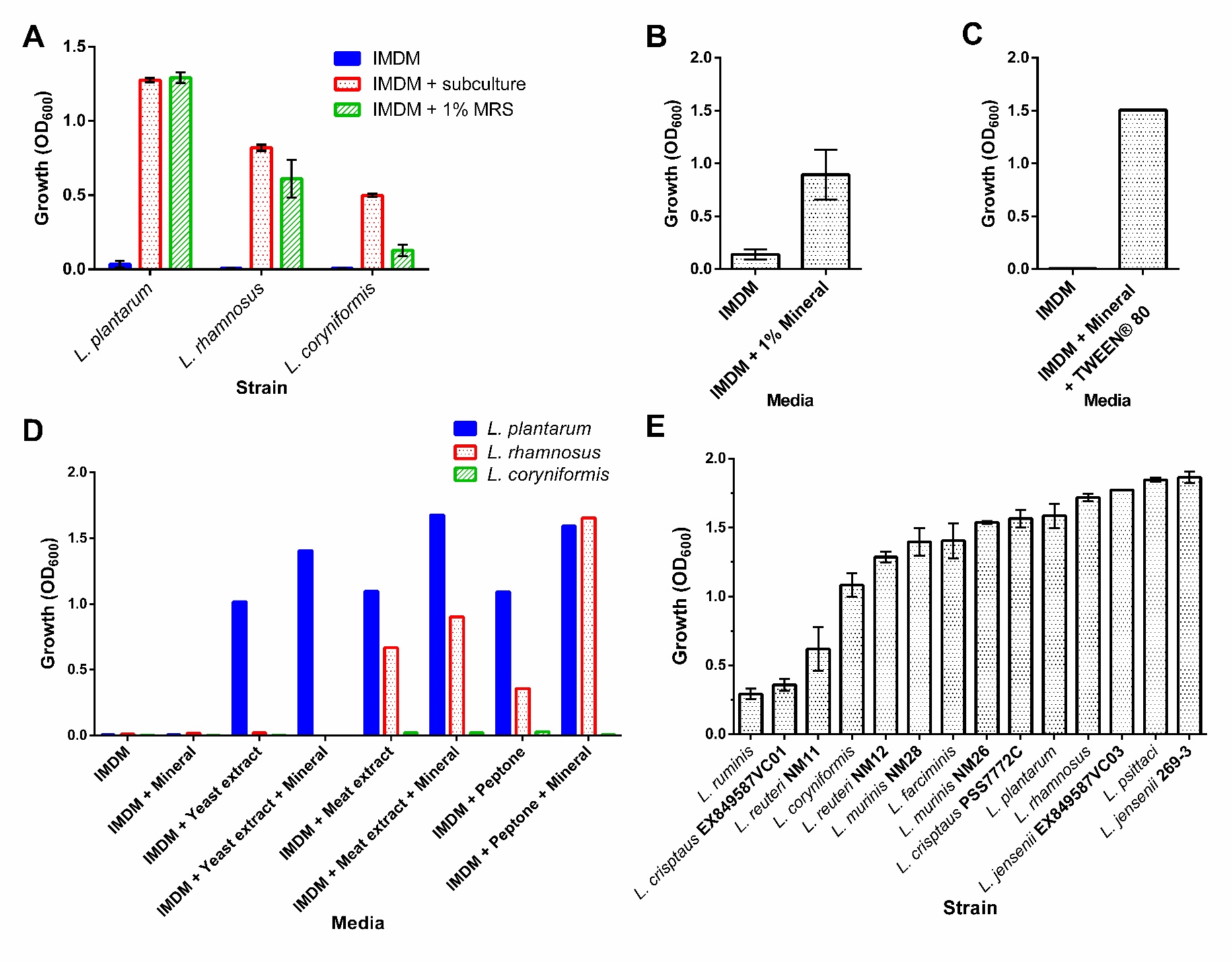


**Figure S2: Lactobacilli growth assays used to develop SLIM.** For strains used, see Tables 3 and S4. **A)** Growth of *L. plantarum, L. rhamnosus,* and *L. coryniformis* in IMDM, IMDM with 1% liquid bacterial culture from MRS, or IMDM with 1% MRS. All media was inoculated with colonies from agar plates. Growth in 4 mL after 24 hours incubation at 37 °C. n = 2, mean ± SD. **B)** Growth of *L. plantarum* in IMDM or IMDM with 1% trace mineral supplement. Growth in 4 mL after 48 hours incubation at 37 °C. n = 2, mean ± SD. **C)** Growth of *L. plantarum* in IMDM or IMDM with 1% trace mineral supplement and 0.1% TWEEN® 80. Growth in 4 mL after 24 hours incubation at 37 °C. n = 1. **D)** Growth of *L. plantarum, L. rhamnosus,* and *L. coryniformis* in IMDM with MRS components, with or without 1% trace mineral supplement. MRS components were yeast extract (4 g/L), meat extract (8 g/L), or peptone (10 g/L). Growth in 4 mL after 24 hours incubation at 37 °C. n = 1. **E)** Growth of vaginal and non-vaginal lactobacilli in IMDM with 10 g/L peptone, 1% trace mineral supplement, and 0.1% TWEEN® 80. Growth in 4 mL after 24 hours incubation at 37 °C. n = 2, mean ± SD.

**
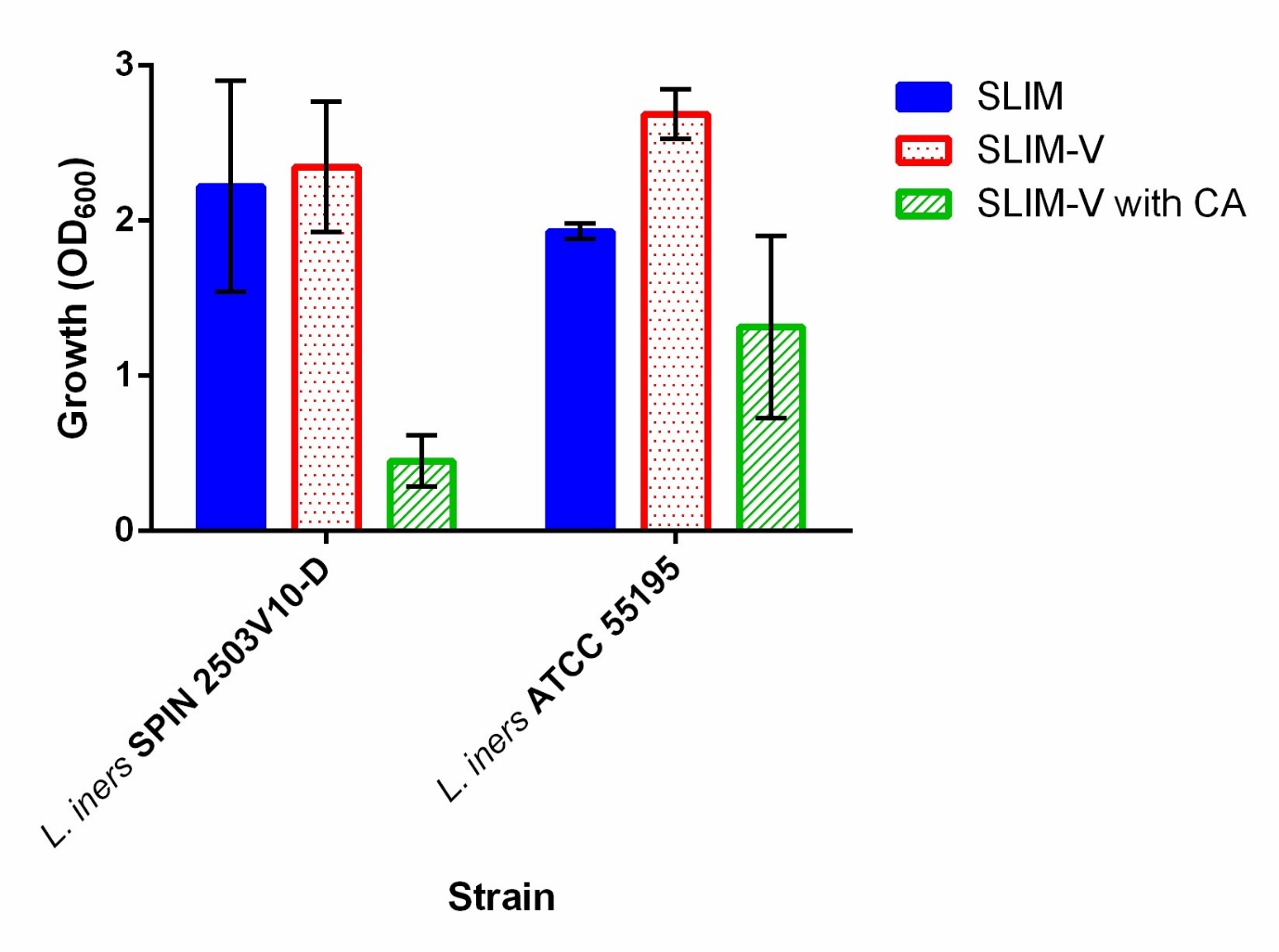
**

**Figure S3: Growth of *L. iners* and in SLIM, SLIM-V, and SLIM-V with casamino acids.** “SLIM-V with CA” has casamino acids and a nucleotide supplement in lieu of peptone. Growth in 2 mL of media after 24 hours incubation at 37 °C. OD_600_ measured using cuvettes (Sarstedt, Nümbrecht, Germany, 67.742) in Ultrospec 3100 Pro (Amersham Biosciences, Piscataway, NJ). n = 3, mean ± SD.

**
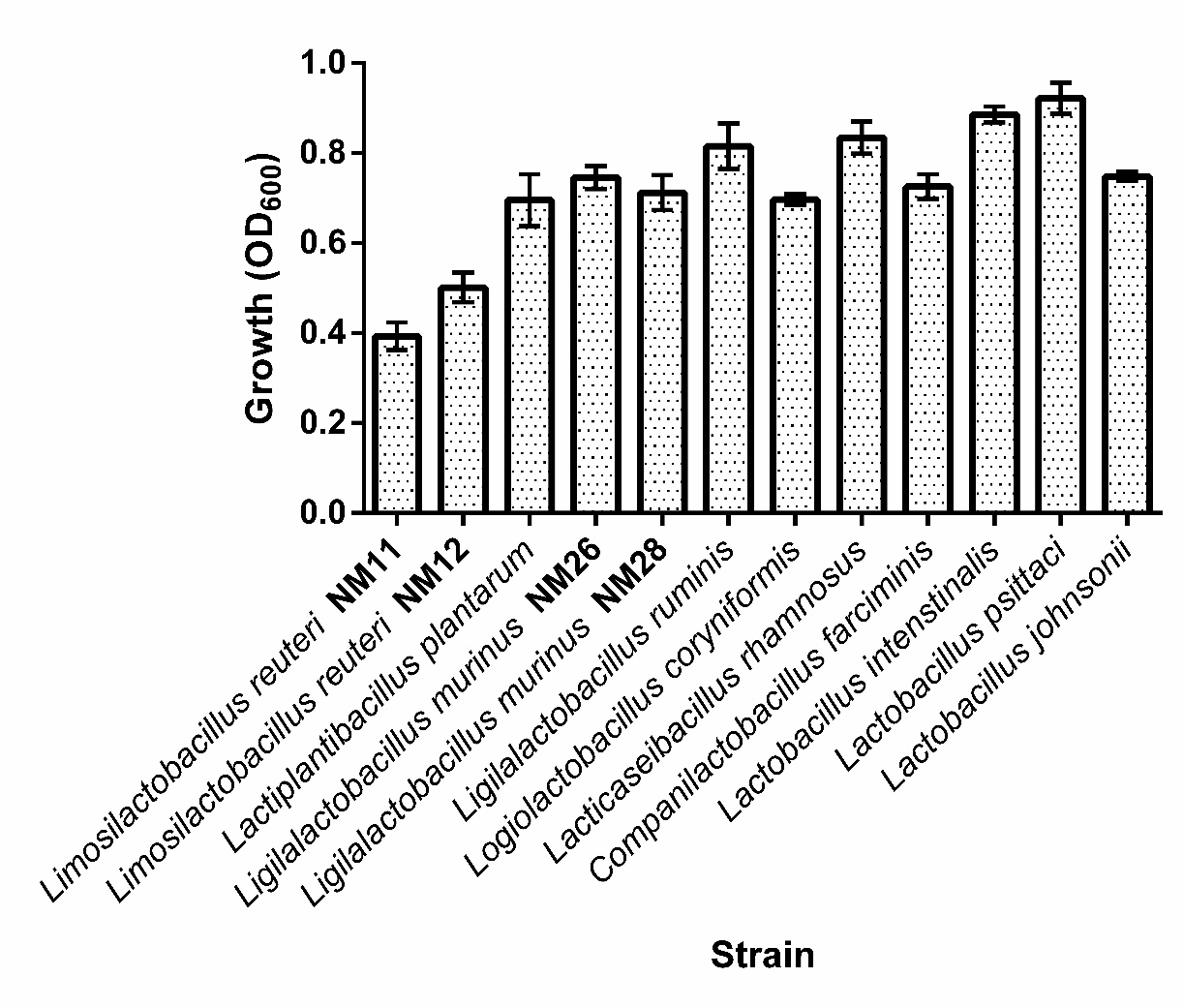
**

**Figure S4: Growth of non-vaginal lactobacilli in SLIM.** Growth in 5 mL after 24 hours incubation. OD_600_ measured using SpectraMax Plus 384 Microplate Reader (Molecular Devices, San Jose, CA). Average of 3 media blanks subtracted. n = 3, mean ± SD.


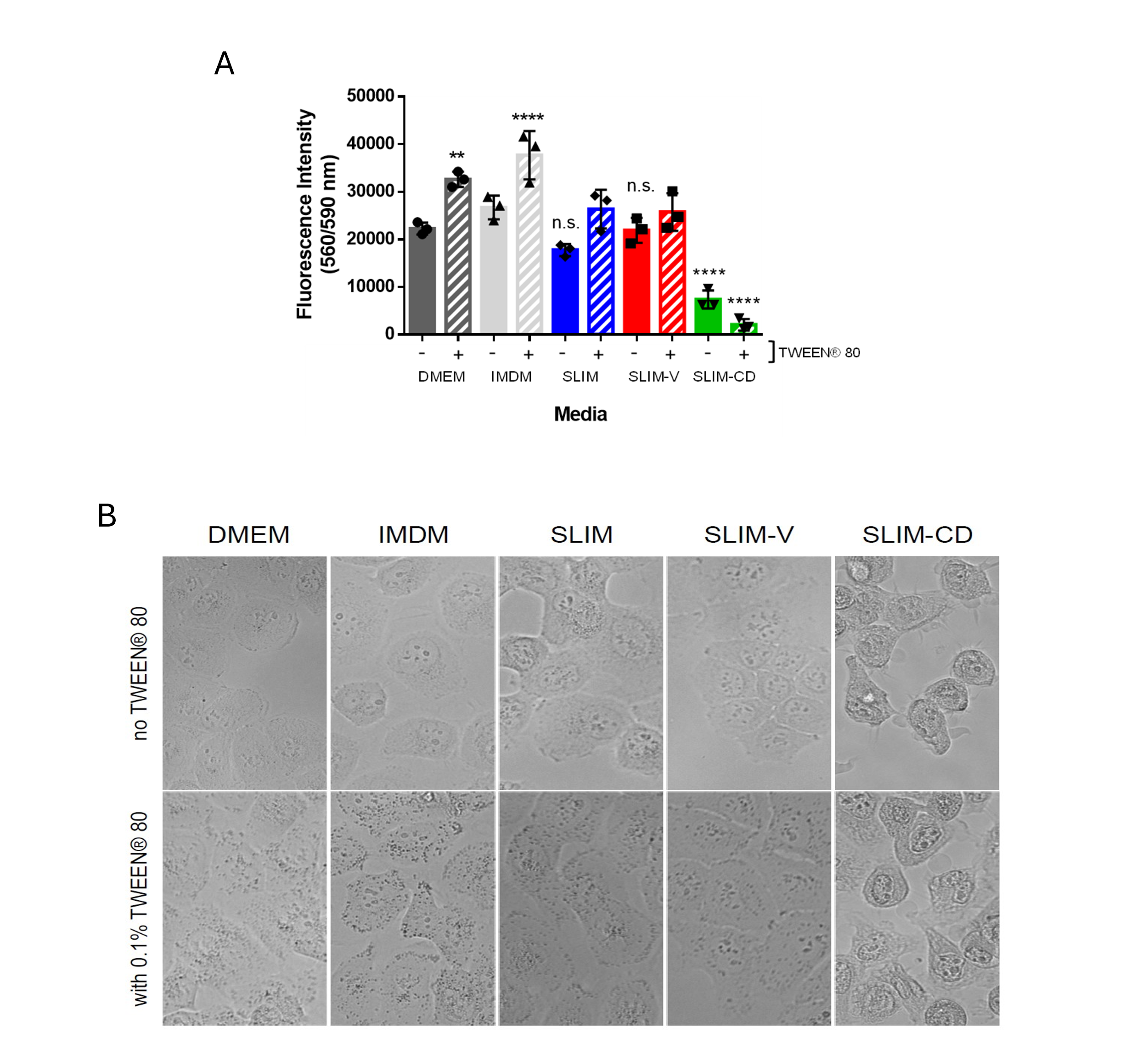


**Figure S5: Viability of HEC-1-B cells in SLIM formulations. A)** Resazurin metabolic measure of cell viability where the fluorescence intensity of metabolized resazurin (resorufin) produced by live cells is proportional to cell viability. Background fluorescence of the media-only controls is subtracted from the fluorescence intensity of the appropriate sample wells. Bars represent the mean ± SD; n=3. Statistical significance was calculated using one-way ANOVA, **p < 0.01. Not significant (n.s.) **B)** Representative light microscopy images of HEC-1-B cells in varying media formulations after 3 hours of incubation.


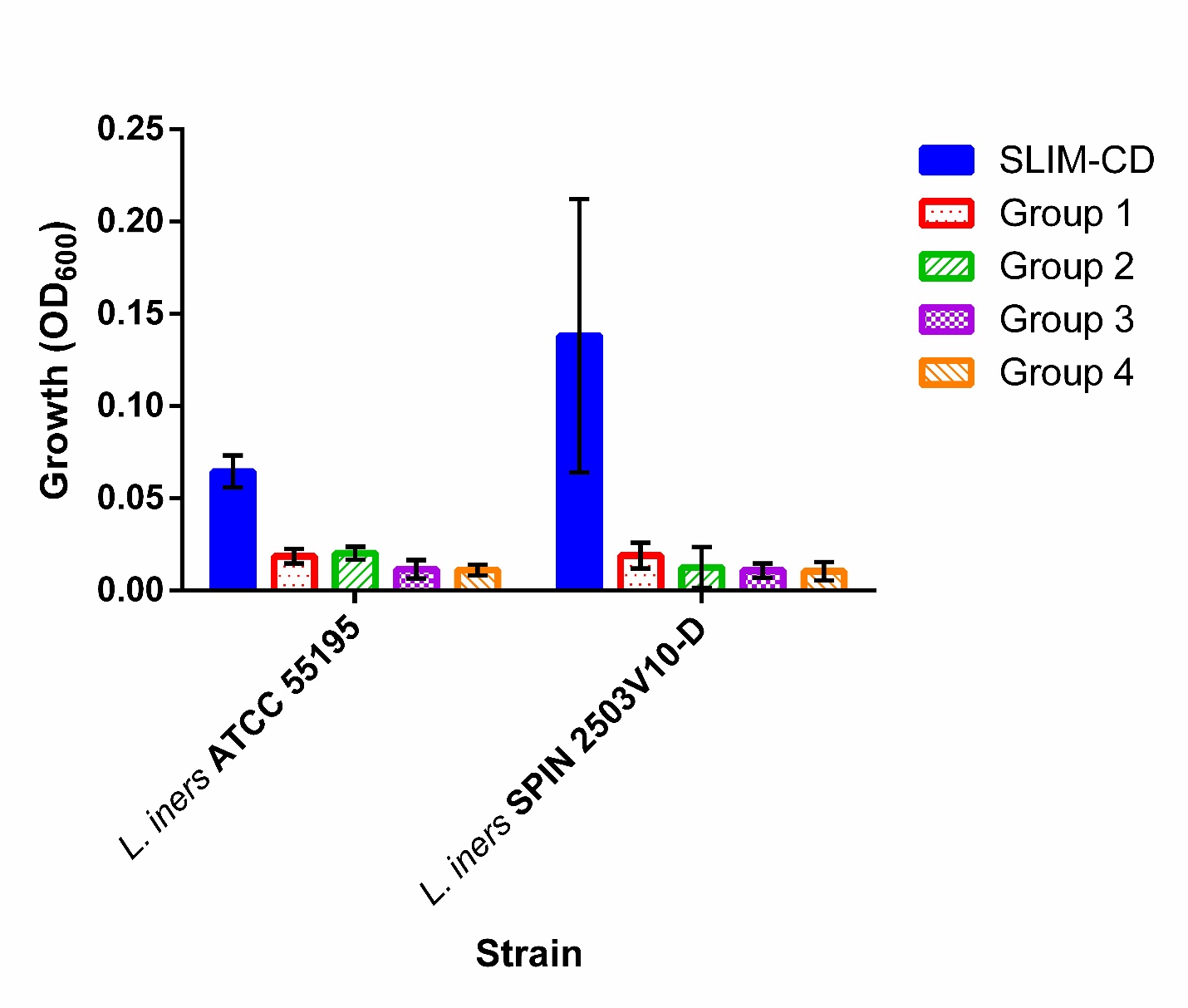


**Figure S6: Growth of *L. iners* in SLIM-CD with subsets of amino acids.** Growth in 100 µL after 24 hours of incubation at 37 °C. Media was inoculated with 1 µL liquid culture in SLIM (4 mL) that had been incubated for 36 hours. Media base was SLIM-CD without amino acids. For each group, specific amino acids were then added at concentrations used in SLIM-CD. Group 1 contains glutamic acid, leucine and proline. Group 2 contains lysine, serine, aspartic acid, tyrosine, and valine. Group 3 contains isoleucine, threonine, arginine, glycine, and phenylalanine. Group 4 includes alanine, histidine, methionine, and tryptophan. OD_600_ measured using SpectraMax Plus 384 Microplate Reader (Molecular Devices, San Jose, CA). Average of 2 media blanks subtracted. n = 3, mean ± SD.

**
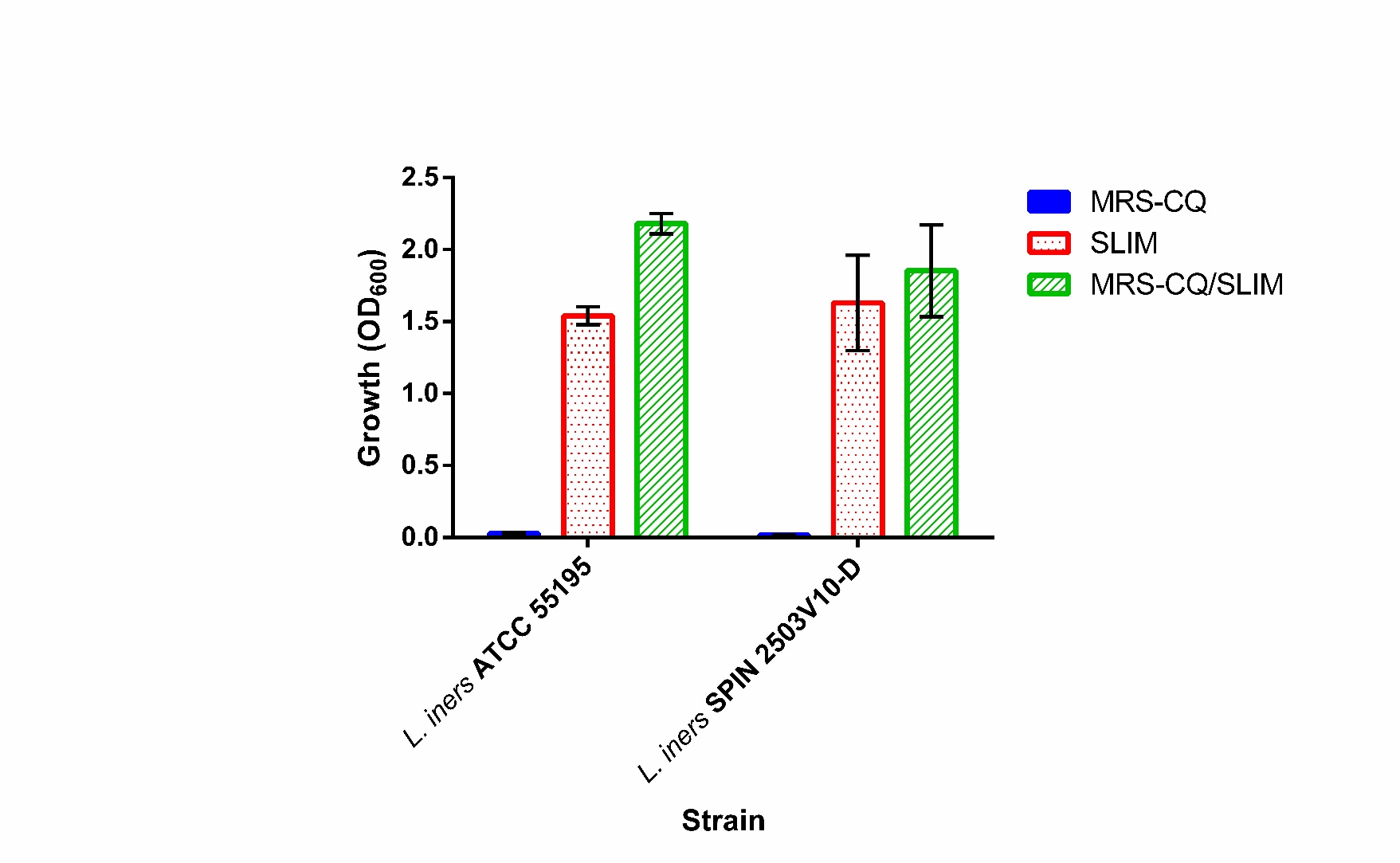
**

**Figure S7: *L. iners* growth in mixture of MRS-CQ and SLIM.** Growth in MRS-CQ, SLIM, or 1:1 mixture (*MRS-CQ/SLIM*). Here MRS-CQ was prepared using NutriSelect® Basic MRS (Sigma-Aldrich, St. Louis, MO, 69966-500G) rather than the BD Difco MRS. Growth in 4 mL of media after 48 hours incubation. OD_600_ measured using cuvettes (Sarstedt, Nümbrecht, Germany, 67.742) in an Ultrospec 3100 Pro (Amersham Biosciences, Piscataway, NJ). n = 3, mean ± SD.

**Table S1: Comparison of simulated vaginal fluid and SLIM components.** Simulated vaginal fluid recipe from Owen & Katz^28^. SLIM concentrations based on IMDM and trace mineral supplement concentrations, as components of peptone are unknown. (–) indicates absent from SLIM.

| **Simulated Vaginal Fluid** | | **Concentration in SLIM (g/L)** | **Reason for Exclusion from SLIM-V** |
| --- | --- | --- | --- |
| **Component** | **Concentration (g/L)** |  |  |
| NaCl | 3.51 | 4.5 | Similar concentration |
| KOH | 1.40 | 0.33 (KCl)  0.000076 (KNO_3_) | Similar concentration |
| Ca(OH)_2_ | 0.222 | 0.166 (CaCl_2_) | Similar concentration |
| Glucose | 5.0 | 4.5 | Similar concentration |
| Bovine serum albumin | 0.018 | - | Peptone provides peptide source |
| Lactic acid | 2.00 | - | Produced by *Lactobacillus* |
| Acetic acid | 1.00 | - | **Included** |
| Glycerol | 0.16 | - | **Included** |
| Urea | 0.4 | - | **Included** |

**Table S2: Composition of NYC III broth**^29^ **supplemented with 10% horse serum.** Recipe for 400 mL. Horse serum added after autoclaving, once media had cooled.

| **Compound** | **Amount** | **Supplier** | **CAT #** |
| --- | --- | --- | --- |
| ddH_2_O | 360 mL | - | - |
| HEPES, 1 M | 6.72 mL | BioShop | HEP001.500 |
| Bacto proteose peptone | 6 g | Gibco | 211684 |
| Sodium chloride | 2 g | BioShop | SOD002.205 |
| D-(+)-Glucose | 2 g | Sigma | G8270-1KG |
| Bacto yeast extract | 1.5 g | BD Difco | 212750 |
| Horse serum, heat inactivated | 40 mL | Gibco | 26050088 |

**Table S3: Primers for identification of *L. iners*, *L. crispatus*, *L. gasseri* and *Gardnerella* isolates.** Primers target genes unique to each species/genus and conserved among strains of that species/genus.

| **Species** | **Target Gene** | **Forward** | **Reverse** | **Source** |
| --- | --- | --- | --- | --- |
| *L. iners* | Inerolysin | TACTAAGCCTG  CACAAGC | TGCATCAAATA  CATCACCTGG | This study |
| *L. crispatus* | Hypothetical protein | TGGCGAAGAG  ACACCAATATC | TGACGTAACG  CATGATGAAT | You & Kim, 2020^42^ |
| *L. gasseri* | 6-phospho-beta-galactosidase | GGACTCGTATCTTTCCTAATGG | TAAGGGAACGAATGTTTATGGG | This study |
| *Gardnerella* sp. | Elongation factor Tu | TCCCAACCCCA  ACTCACGATCTT | NCGCAAACCAAC  NATCTCAACTGG | Balashov *et al.,* 2014^43^ |

**Table S4: Non-vaginal lactobacilli with growth supported in SLIM.** See Figure S4.

| **Species** | **Strain** | **Source** |
| --- | --- | --- |
| *Limosilactobacillus reuteri* | NM11 | Collection of Inflammation-Associated Mouse Intestinal Bacteria |
| *Limosilactobacillus reuteri* | NM12 | Collection of Inflammation-Associated Mouse Intestinal Bacteria |
| *Lactiplantibacillus plantarum* | DSM 20174 | Pickled cabbage |
| *Ligilalactobacillus murinus* | NM26 | Collection of Inflammation-Associated Mouse Intestinal Bacteria |
| *Ligilalactobacillus murinus* | NM28 | Collection of Inflammation-Associated Mouse Intestinal Bacteria |
| *Ligilalactobacillus ruminis* | DSM 20403 | Bovine rumen |
| *Logiolactobacillus coryniformis* | DSM 20001 | Silage |
| *Lacticaseibacillus rhamnosus* | LMS2-1 | Human gastrointestinal tract |
| *Companilactobacillus farciminis* | DSM 20184 | Sausage |
| *Lactobacillus intenstinalis* | NM61 | Collection of Inflammation-Associated Mouse Intestinal Bacteria |
| *Lactobacillus psittaci* | DSM 15354 | Lung of parrot |
| *Lactobacillus johnsonii* | NM60 | Collection of Inflammation-Associated Mouse Intestinal Bacteria |

**References**

28. Owen, D. H. & Katz, D. F. A vaginal fluid simulant. *Contraception* **59**, 91–95 (1999).

42. You, I. & Kim, E. B. Genome-based species-specific primers for rapid identification of six species of Lactobacillus acidophilus group using multiplex PCR. *PLoS One* **15**, e0230550 (2020).

43. Balashov, S. V., Mordechai, E., Adelson, M. E. & Gygax, S. E. Y. 2. Identification, quantification and subtyping of Gardnerella vaginalis in noncultured clinical vaginal samples by quantitative PCR. *J. Med. Microbiol.,* **63**, 162–175.
